# Measurement of Head Circumference: Implications for Microcephaly Surveillance in Zika-Affected Areas

**DOI:** 10.3390/tropicalmed6010005

**Published:** 2020-12-29

**Authors:** Emily W. Harville, Van T. Tong, Suzanne M. Gilboa, Cynthia A. Moore, Maria Luisa Cafferata, Jackeline Alger, Luz Gibbons, Carolina Bustillo, Allison Callejas, Mario Castillo, Jenny Fúnes, Jorge García, Gustavo Hernández, Wendy López, Carlos Ochoa, Fátima Rico, Heriberto Rodríguez, Concepción Zúniga, Alvaro Ciganda, Candela Stella, Giselle Tomasso, Pierre Buekens

**Affiliations:** 1 Department of Epidemiology, Tulane School of Public Health and Tropical Medicine, New Orleans, LA 70112, USA; pbuekens@tulane.edu; 2 Division of Birth Defects and Infant Disorders, National Center on Birth Defects and Developmental Disabilities, Centers for Disease Control and Prevention, Atlanta, GA 30333, USA; vct2@cdc.gov (V.T.T.); suz0@cdc.gov (S.M.G.); cam0@cdc.gov (C.A.M.); 3 Instituto de Efectividad Clinica y Sanitaria, Buenos Aires 1414, Argentina; marialuisa.cafferata@gmail.com (M.L.C.); luzgibbons@gmail.com (L.G.); cstella@iecs.org.ar (C.S.); 4 Unidad de Investigación Clínica y Epidemiológica, Montevideo 11600, Uruguay; aciganda@gmail.com (A.C.); gtomasso@unicem-web.org (G.T.); 5 Departamento de Laboratorio Clínico, Hospital Escuela, Tegucigalpa 11101, Honduras; jackelinealger@gmail.com (J.A.); jalgar62_84@yahoo.com.ar (J.G.); wlopez36@hotmail.com (W.L.); 6 Instituto de Enfermedades Infecciosas y Parasitología Antonio Vidal, Tegucigalpa 11101, Honduras; concepcionzuniga@gmail.com; 7 Unidad de Investigación Científica, Facultad de Ciencias Médicas, Universidad Nacional Autónoma de Honduras, Tegucigalpa 11101, Honduras; 8 Departamento de Ginecología y Obstetricia, Hospital Escuela, Tegucigalpa 11101, Honduras; mcbu1502@yahoo.com; 9 Departamento de Ginecología y Obstetricia, Facultad de Ciencias Médicas, UNAH, Tegucigalpa 11101, Honduras; 10 Servicio de Neonatología, Departamento de Pediatría, Hospital Escuela, Tegucigalpa 11101, Honduras; amariecs1981@gmail.com (A.C.); mariocastillo26@yahoo.com (M.C.); jennylagosfunes@yahoo.com (J.F.); 11 Departamento de Pediatría, Facultad de Ciencias Médicas, Universidad Nacional Autónoma de Honduras, Tegucigalpa 11101, Honduras; ricourrea7@gmail.com; 12 Departamento de Pediatría, Hospital de Especialidades San Felipe, Tegucigalpa 11101, Honduras; ghernandezbustillo@yahoo.es; 13 Servicio de Maternidad, Hospital de Especialidades San Felipe, Tegucigalpa 11101, Honduras; 14 Departamento de Pediatría, Hospital Escuela, Tegucigalpa 11101, Honduras; 15 Sub-Dirección, Hospital de Especialidades San Felipe, Tegucigalpa 11101, Honduras; mmfhrodriguez@yahoo.com.mx; 16 Departamento de Vigilancia de la Salud, Hospital Escuela, Tegucigalpa 11101, Honduras

**Keywords:** microcephaly, measurement, Central America, Zika virus, neonate

## Abstract

Worldwide recognition of the Zika virus outbreak in the Americas was triggered by an unexplained increase in the frequency of microcephaly. While severe microcephaly is readily identifiable at birth, diagnosing less severe cases requires comparison of head circumference (HC) measurement to a growth chart. We examine measured values of HC and digit preference in those values, and, by extension, the prevalence of microcephaly at birth in two data sources: a research study in Honduras and routine surveillance data in Uruguay. The Zika in Pregnancy in Honduras study enrolled pregnant women prenatally and followed them until delivery. Head circumference was measured with insertion tapes (SECA 212), and instructions including consistent placement of the tape and a request to record HC to the millimeter were posted where newborns were examined. Three indicators of microcephaly were calculated: (1) HC more than 2 standard deviations (SD) below the mean, (2) HC more than 3 SD below the mean (referred to as “severe microcephaly”) and (3) HC less than the 3rd percentile for sex and gestational age, using the INTERGROWTH-21st growth standards. We compared these results from those from a previous analysis of surveillance HC data from the Uruguay Perinatal Information System (Sistema Informático Perinatal (SIP). Valid data on HC were available on 579 infants, 578 with gestational age data. Nine babies (1.56%, 95% CI 0.71–2.93) had HC < 2SD, including two (0.35%, 95% CI 0.04–1.24) with HC < 3SD, and 11 (1.9%, 95% CI, 0.79–3.02) were below the 3rd percentile. The distribution of HC showed strong digit preference: 72% of measures were to the whole centimeter (cm) and 19% to the half-cm. Training and use of insertion tapes had little effect on digit preference, nor were overall HC curves sufficient to detect an increase in microcephaly during the Zika epidemic in Honduras. When microcephaly prevalence needs to be carefully analyzed, such as during the Zika epidemic, researchers may need to interpret HC data with caution.

## 1. Introduction

The major indicator that Zika virus (ZIKV) infection during pregnancy was affecting fetal brain development was an increase in microcephaly [1]. Severe microcephaly is readily identifiable at birth, but less severe cases require comparison of head circumference (HC) measurement to growth charts. There is no single clinical definition for microcephaly based on HC; widely-used definitions include HC 2 standard deviations (SDs) below the mean (3 SD for severe microcephaly) or below the 3rd percentile for sex and gestational age [2]. Percentile- and SD-based definitions are inherently problematic because, by definition, some proportion of the population will fall below the cut-off, even if completely healthy. However, even aside from this issue, the prevalence of microcephaly in surveillance data indicates that stricter definitions, or additional criteria, are generally used in clinical diagnosis. Population estimates of microcephaly were between 0.3 and 12 per 10,000 prior to the Zika epidemic [2,3,4,5,6,7], and in areas unaffected by Zika [8], substantially below the 15–300 per 10,000 that would be expected mathematically (3rd percentile or <3 SD below the mean). Comparing routinely collected data (the Brazilian Live Birth Information System) to those collected by trained research personnel suggested that microcephaly was substantially underreported, perhaps 90% of the time [3]. 

Accurate measurement of HC is challenging. A measuring tape must be placed precisely at the widest possible circumference of the head (the broadest part of the forehead above the eyebrow, above the ears and at the most prominent part of the back of the head [9]), and this can be difficult with a squirmy infant. INTERGROWTH, a global project to define standards for fetal and infant growth [10], recommends that two people assist in the measurement, one holding the infant on his or her lap, a procedure that is not always possible in a busy delivery room where the primary concern is likely to be assuring the health of mother and baby [11]. In addition, small degrees of tightening of the measuring tape can affect the measurement. One study showed that applying the tape tightly or loosely can induce a difference of 0.4 SD (or 0.4–0.5 cm) [12], while another found an inter-observer variability over 1 cm in 9% of the measurements [13]. Insertion tapes are regarded as more standardized and reliable than standard tape measures, and are used by the U.S. National Health and Nutrition Examination Survey (NHANES) [14,15], but the evidence that they improve measurement quality is limited. Studies have found parents can be trained to measure infant anthropometry, including HC, with good reliability and validity, although the error of measurement was 0.6 cm [16]. Since growth charts are to the millimeter, this still allows for substantial error.

Surveillance for microcephaly, generally or to assess trends in Zika-related illness, therefore becomes difficult. Multiple definitions of microcephaly make it challenging to compare trends over time or across countries [17]. Detection may be impacted by increased awareness due to the Zika epidemic [18,19]. The combination of measurement error and low prevalence means detecting a signal may be possible only in very severe outbreaks. 

In this analysis, we examine HC measured values and digit preference in those values, and, by extension, the prevalence of microcephaly at birth in two groups. One is within a study in Honduras, where clinicians were part of a large-scale training and used insertion tape consistently for HC measures. We compare these data collected as part of our research study in Honduras to routine surveillance data from Uruguay [8].

## 2. Materials and Methods 

**Figure 1:**
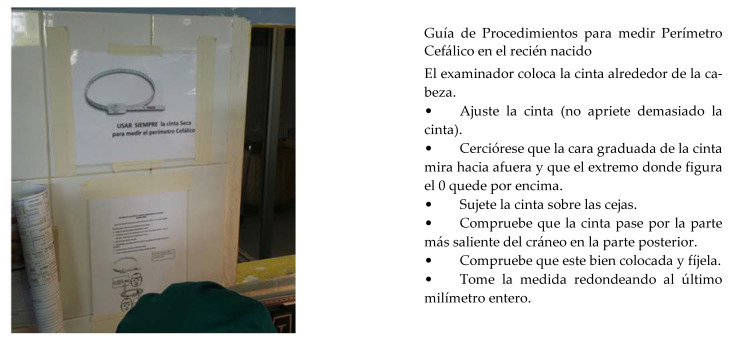

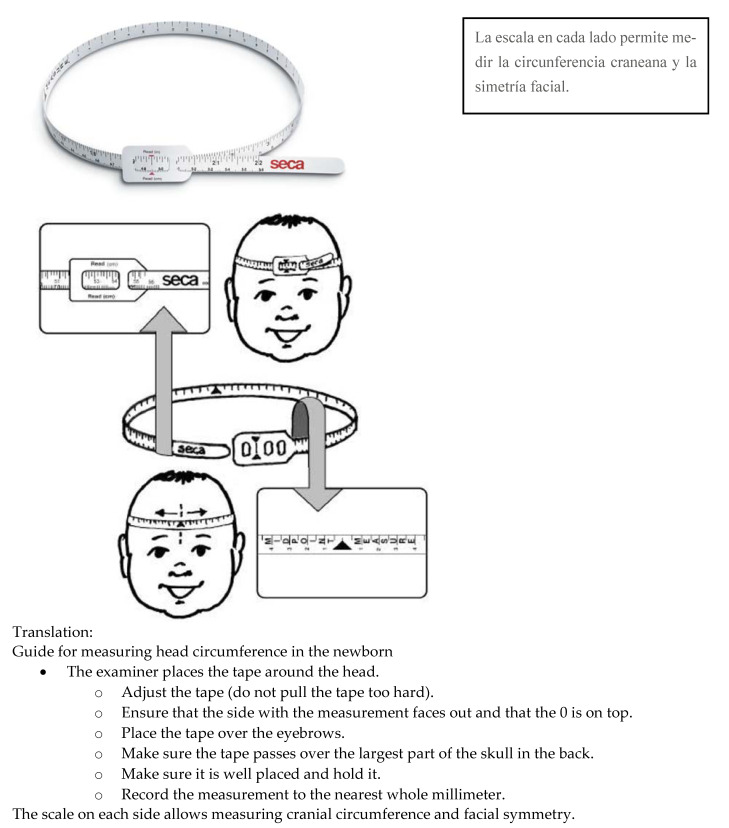
Instructions for use of SECA 212 insertion tapes posted in the room where newborns were measured, Zika in Pregnancy in Honduras (ZIPH) (2016). Hospital Escuela, Tegucigalpa, Honduras. Sign translates, “Always use the Seca tape to measure head circumference.”

We conducted a prospective pregnancy cohort study in Honduras in 2016 to study ZIKV infection: The Zika in Pregnancy in Honduras (ZIPH) study; details have been published previously [20]. We enrolled pregnant women at their first prenatal visit and followed them up until delivery. At the time of enrollment, women were interviewed to collect contact information for additional follow-up, sociodemographics, and symptoms of ZIKV infection. Care was taken to measure HC at birth accurately using best practices: insertion tapes (SECA 212) were provided, and instructions were posted in the room where newborns were measured (Figure 1), including instructions on the location of the tape relative to the child’s head and consistent placement of the markings and a specific request to record to the last whole millimeter. The tapes were donated to Neonatology Service at Hospital Escuela on 2 June 2016, and to Neonatology Service at Hospital San Felipe on 4 July 2016, three to seven weeks before the study started. Once the tapes were received, face-to-face training began. Starting in August 2016, INTERGROWTH-21st measures and training (online module on assessing newborn size by anthropometry) were promoted in the pediatrics department [21]. Initial dissemination was to pediatricians and neonatologists, followed by students. Health providers (medical students and residents) measuring the newborns were trained on a regular basis, including both face-to-face and online training, using the INTERGROWTH-21st materials. Supervising neonatologists and pediatricians monitored measurement during shifts and reviewed clinical charts on an ongoing basis. Starting in January 2017, certification was incorporated into the academic course for 7th year medical students (interns) and pediatrics residents. Between January and June 2017, 14 trainings were conducted with 192 students; over 95% of the students received the certification before beginning rotations. In addition, at each student rotation change (monthly or fortnightly) there was a verification of skills. For each newborn, the measurement was performed twice, at birth and at discharge or at 24 h. Only the birth measurement, which was part of the research protocol, is analyzed here.

We compared these results to those from a previous analysis of surveillance data in South America. We analyzed HC data from the Uruguay Perinatal Information System (Sistema Informático Perinatal (SIP) [8]. Briefly, this system includes data collected clinically as part of routine care, including the perinatal clinical record, delivery card, and neonatal hospitalization data [22]. The recommendation by the Ministry of Health is that clinicians use a flexible and inelastic tape to measure HC [23]. The HC variable allows for recording as centimeters with one decimal place. The Uruguay dataset included 261,330 live births, and for the purposes of this analysis, comparison was limited to plausible values, operationalized as those within 5 SD of the mean (n = 255,919, 99% of those with head circumference measures).

Microcephaly at birth was defined as HC more than 2SD below the mean, more than 3SD below the mean (severe microcephaly), or below the 3rd percentile for sex and gestational age (GA), using the INTERGROWTH-21st charts [8,10]. Confidence intervals for proportions were calculated using standard errors based on a binomial distribution. GA was estimated most commonly by last menstrual period (516/626, 82%); otherwise by ultrasound (90/626, 14%), clinical examination, or Capurro scores [24]. R and SAS 9.4 were used for data analysis. This study was reviewed and approved by the Tulane University and the Faculty of Medical Sciences, Universidad Nacional Autónoma de Honduras institutional review boards.

## 3. Results

In the ZIPH study, 667 women were enrolled in 2016 and 645 followed until the end of their pregnancy, including 600 livebirths, 36 early losses, and 9 stillbirths. Valid data on HC were available for 579 and for HC and GA on 578 (most of those missing data were delivered at other hospitals or their charts could not be located). Nine babies (1.56%, 95% CI 0.71–2.93) had HC more than 2SD below the mean, including two (0.35%, 95% CI 0.04–1.24) with HC more than 3SD below the mean, and 11 (1.9%, 95% CI, 0.79–3.02) below the 3rd percentile (Table 1).

**Figure 2:**
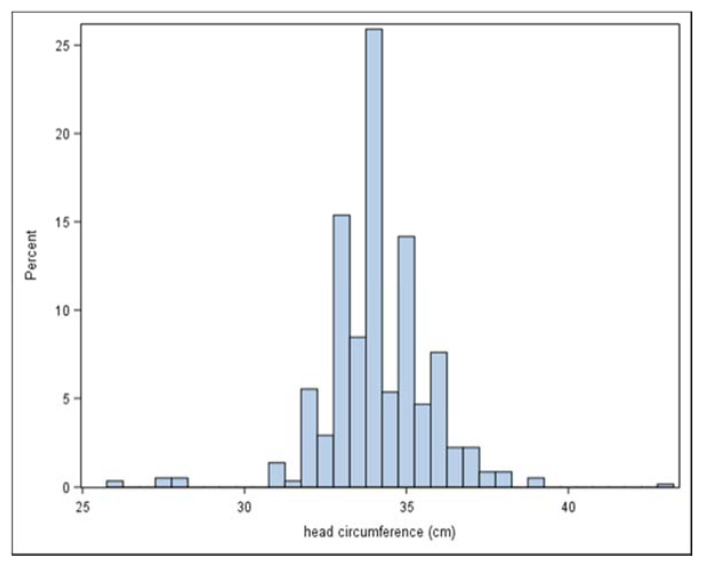
Distribution of head circumference at birth in the Zika in Pregnancy in Honduras (ZIPH) cohort (n = 579).

The distribution of HC (Figure 2, Table 2) showed strong digit preference, despite the training and use of insertion tapes for HC measurement. 72% of recorded measures were to the whole cm, with an additional 19% to the half-cm. No recorded measures were to 0.1, and only a small number to other mm. The two cases of severe microcephaly associated with ZIKV infection were not sufficient to meaningfully increase the overall rate of microcephaly in the Honduras study during the ZIKV outbreak (Table 1).

**Figure 3:**
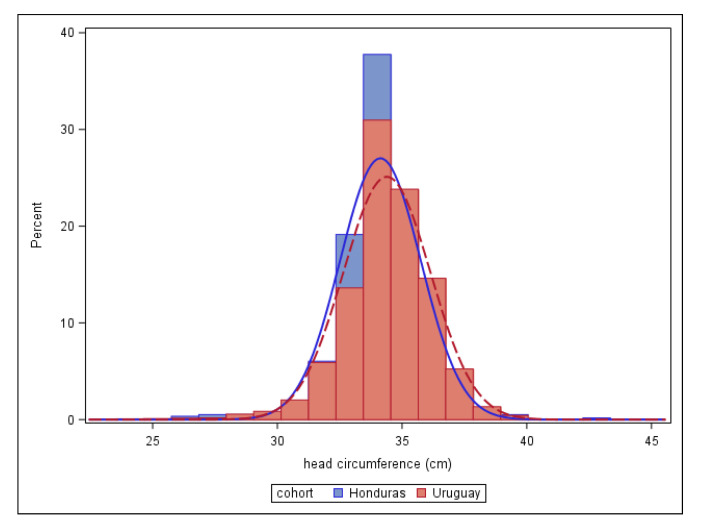
Distribution of head circumference in the Zika in Pregnancy in Honduras cohort (2016) and the Uruguay SIP surveillance system (2010–2015).

When compared to a previous analysis using surveillance data in Uruguay [8], mean and median HC were slightly lower (Figure 3). Mean z-score relative to INTERGROWTH-21st standards in the ZIPH cohort was 0.55 and median z-score was 0.53, while in the Uruguay data, the mean z-score was 0.77 and the median was 0.84. 

## 4. Discussion

In this study, training and use of insertion tapes had little effect on digit preference, nor were overall HC curves sufficient to detect an increase in microcephaly during the Zika epidemic in Honduras. We also compared this with a previous analysis we had done on routine HC measurements in a non-Zika-affected country. Despite the different countries, clinical settings, and degree of focus on and training in HC measurement, several results were similar across the two settings. When microcephaly was considered by SD definitions, proportions in each group were lower than mathematically expected (similar to the Uruguay analysis), though the calculated confidence intervals for the proportions were consistent with those percentiles. Mean and median HC were slightly lower in Honduras than in Uruguay, but the left tail of the distribution was not noticeably thicker (Figure 3). This is despite the fact that 2016 marked the peak for Zika infection in Honduras, while the epidemic never spread to Uruguay. We also found similar results in the two cohorts in terms of digit preference, with the vast majority being measured to the cm or half-cm (Table 2). It appears the provision of and training on SECA 212 tapes had little effect on the tendency to measure to the whole centimeter, although rounding to the half-centimeter was diminished a little (19% in Honduras compared to 25% in Uruguay). A study of measurement protocols in children under 5 in Atlanta, Georgia also found digit preference for HC, though much less to decimal places 0 and 5 [25]. However, even in the rigorously standardized INTERGROWTH study, technical error of measurement was still 0.3−0.4 cm [26]. Our study is limited by the small sample size and the lack of pre-training measures.

Simulations indicate that surveillance data are generally insufficient to estimate the gestational-age-specific risk of microcephaly, especially given possible changes in reporting that might be caused by an epidemic [27,28]. Birth defect surveillance data from Colombia did indicate a peak in microcephaly (based on diagnosis code) corresponding with the Zika epidemic; however, very large numbers would be needed to detect the observed differences in prevalence (13 cases per 10,000 livebirths during the outbreak, vs. 8 per 10,000 before and 11 per 10,000 after) [29]. In addition, percentile-based definitions produce a high degree of false positives when compared to truly pathological cases; simulation studies under realistic scenarios estimate <5% positive predictive value [27]. 

Several studies show that, regardless of efforts to standardize, measurement of HC is not necessarily accurate or precise. A 2018 survey of U.S. nurses showed that a minority of respondents were recording HC to the nearest 0.1 cm, while a majority measured 0.5 cm or larger increments [30]. A Chinese study found that test-retest reliability for HC was good, with correlations > 0.95, but the mean interobserver difference was 0.4 cm [31]. Studies of measurement of growth in children indicate that even basic measurements like height are often inaccurate, although training can improve them [32]. Measurement protocols also differ even when rigorously standardized—the U.S. Centers for Disease Control and Prevention’s recommendation for the Zika epidemic was to measure three times and take the largest measurement [9], while NHANES requires only a single measure [15]. Because of the limited reliability, other methods of assessing HC have been sought, including digital calipers that limit stretching [33], and use of biparietal diameter instead of circumference [34]; however, limited availability and required maintenance of appropriate instruments, as well as lack of sex and GA standards for biparietal diameter in most populations might be challenges for widespread adoption of these methods. As training on the SECA tapes was not successful in correcting issues identified with the measurement of head circumference in hospital settings, it is probable that precise head circumference measurement in the delivery room is an unrealistic goal. Our findings, showing some improvement in diminishing half-centimeter measurements, indicate that there might be the need for additional or regular refresher training. Other tools and procedures, such as visual reminders in the form of additional posters in the nursery or other parts of the wards, short instructional videos, and periodic review of head circumference measurements by nursery staff to look for these patterns, could also be helpful. Ultimately, the development of technology that is less prone to user variation is needed.

We conclude that researchers may want to interpret HC data with caution. Under circumstances when microcephaly prevalence needs to be carefully analyzed and trends determined, such as the Zika epidemic, researchers might consider developing and evaluating new measurement methods. 

## Figures and Tables

**Table 1:** Description of the Zika in Pregnancy in Honduras (ZIPH) cohort, 2016.

All Women		
	n	%
**Maternal Variables**		
Maternal age in years (n = 667)		
12–19	198	29.7%
20–34	404	60.6%
35 or higher	65	9.8%
Years of education and range (n = 667)		9 (6–12)
**Infant Variables**		
Head circumference at birth (n = 578)		
mean in cm (SD)		34.1 (1.62)
median in cm (IQR)		34.0 (33.0–35.0)
mode in cm, range		34.0 (26–43)
HC more than 2SD below the mean *	9	1.56, 95% CI 0.71–2.93 **
HC more than 3SD below the mean *	2	0.35, 95% CI 0.04–1.24
HC < 3rd percentile *	11	1.90, 95% CI 0.79–3.02

HC, head circumference; SD, standard deviation; IQR, interquartile range. * For sex and gestational age. ** calculated using standard errors based on binomial distribution.

**Table 2:** Distribution of head circumference measures for the Zika in Pregnancy in Honduras Cohort (2016) and the Uruguay Perinatal Information System (Sistema Informático Perinatal, SIP) Surveillance System (2010−2015).

	ZIPH Cohort		Uruguay SIP	
Last Digit (Tenths Place)	N	%	N	%
0	419	72.4	188,108	73.5
0.1	0	0.0	102	0.0
0.2	10	1.7	444	0.2
0.3	8	1.4	462	0.2
0.4	5	0.9	435	0.2
0.5	112	19.3	65,196	25.5
0.6	15	2.6	304	0.1
0.7	7	1.2	291	0.1
0.8	2	0.3	432	0.2
0.9	1	0.2	145	0.1
